# Planned Reoperation after Cardiac Surgery in the Cardiac Intensive Care Unit

**DOI:** 10.31083/j.rcm2403087

**Published:** 2023-03-08

**Authors:** Zhigang Wang, Yubei Kang, Zheyun Wang, Jingfang Xu, Dandan Han, Lifang Zhang, Dongjin Wang

**Affiliations:** ^1^Department of Cardio-thoracic Surgery, Affiliated Drum Tower Hospital, Medical School of Nanjing University, 210008 Nanjing, Jiangsu, China; ^2^Department of Nephrology, Nanjing Drum Tower Hospital Clinical College of Nanjing University of Chinese Medicine, 210023 Nanjing, Jiangsu, China; ^3^Department of Psychiatry, The First Affiliated Hospital, Zhengzhou University, 450001 Zhengzhou, Henan, China

**Keywords:** cardiac surgery, reoperation, cardiac intensive care unit, hemorrhagic shock, mortality

## Abstract

**Background:**

Cardiac surgical re-exploration for bleeding is associated with increased morbidity and mortality. Whether to perform these procedures in the operating room (OR) or the Cardiac Intensive Care Unit (CICU) in uncertain. We sought to determine if the location of the reoperation would affect postoperative outcomes when a reoperation for bleeding is required following cardiac surgery.

**Methods:**

Patients who underwent planned cardiac re-explorations for bleeding at our center from January 2019 to December 2021 were retrospectively enrolled in this study. Patient outcomes were compared and analyzed.

**Results:**

Due to hemorrhagic shock, 72 patients underwent planned cardiac re-explorations, including 21 operated in the CICU and 51 in the OR. Within 12 h of the primary operation, 65 re-explorations (90.3%) were performed. The peak Vasoactive-Inotropic Score was 47.0 ± 27.4, systolic blood pressure was 89.4 ± 9.6 mmHg, central venous pressure was 12.1 ± 4.4 ${\mathrm{cmH}}_{2}$O, and the serum lactate was 5.5 ± 4.1 mmol/L prior to the reoperation. Multivariate logistic analysis showed that a reoperation performed in the CICU was not an independent risk factor for the occurrence of major complications. There was no significant difference in mortality between the two groups.

**Conclusions:**

Planned re-exploration for bleeding following open cardiac surgery in the CICU is feasible and safe.

## 1. Introduction

Excessive bleeding after cardiac surgery is a severe postoperative complication that is often accompanied by hemorrhagic shock and can occur in up to 12% of patients [1]. Postoperative bleeding has been associated with increased mortality, prolonged stay in the cardiac intensive care unit (CICU) and higher rates of sternal wound infection (SWI) [2, 3, 4]. Re-exploration for bleeding after open-heart surgery has been conventionally performed in the operating room (OR) except for patients in cardiac arrest who most often undergo surgery immediately in the CICU. Returning patients to OR may delay the operation and may result in additional risks to patients due to OR availability and the need for transportation.

Alternatively, conducting the re-exploration in the CICU allows for a more rapid procedure and can save both hospital and patient resources. However, controversies have been raised in conducting such surgery in the CICU due to the relative non-sterile environment [5]. Two previous reports supported the safety of performing chest re-exploration in the CICU [6, 7]. However, neither compared the postoperative outcomes to procedures performed in the OR. Furthermore, these two studies were limited to short-term outcomes and did not mention the long-term results of postoperative re-exploration conducted in the CICU. Therefore, the purpose of this study was to evaluate and compare outcomes of postoperative mediastinal re-explorations for bleeding following cardiac surgery conducted in the CICU versus the OR.

## 2. Materials and Methods

### 2.1 Patients 

A total of 5726 patients who received open-heart operations at our center between January 2019 and December 2021 were retrospectively screened for this study. Patients who received a planned re-exploration due to bleeding were involved in the study. Patients who received mediastinal re-exploration due to cardiac arrest and cardiac tamponade were excluded. Patients in this cohort urgently needed re-exploration but not emergently. The more urgent cases were re-explored in CICU, while the less urgent patients had time to go to the OR. The CICU and the OR are located at different floors in our center. Therefore, additional time is needed to transfer patients to the OR. The decision as to where the re-exploration was to be performed was made independently by the surgeon who performed the primary heart operation.

The medical records of included patients were retrospectively reviewed. Demographic data, operative characteristics, and patient outcomes were recorded and compared between patients who received re-exploration in the CICU or the OR. The Ethics Committee of Nanjing Drum Tower Hospital approved this study (NO. BL2014004) and waived the need for individual informed consent due to the retrospective nature of the study.

### 2.2 Definitions

Vasoactive drugs were defined as intravenous vasopressors and inotropes administered via continuous infusion, including dobutamine (DOB), dopamine (DOPA), epinephrine (EPI), norepinephrine (NE), phenylephrine (PHEN), vasopressin (VASO) and milrinone (MIL). The peak Vasoactive-Inotropic Score (VIS) was calculated with peak vasoactive drug doses upon ICU admission after cardiac surgery and before reoperation according to following formula (in mcg/kg/min): VIS = DOB + DOPA + (10 × PHEN + MIL) + (100 × [EPI + NE]) + (10,000 × units/kg/min VASO); one VIS unit is considered equivalent to 1 mcg/kg/min of DOB or DOPA or 0.01 mcg/kg/min of EPI or NE [8, 9]. Hemorrhagic shock was defined as a systolic blood pressure <90 mmHg for patients after cardiac surgery. Planned re-exploration was defined as non-emergency surgery conducted in a relative stable hemodynamic condition after fluid resuscitation and use of vasoactive drugs. SWI was diagnosed by clinical signs, intraoperative findings, results of wound healing, blood cultures, and computed tomography imaging. Acute kidney injury (AKI) was diagnosed according to the Kidney Disease Improving Global Outcomes criteria. Major complications were defined as 30-Day all-cause mortality and severe morbidities (SWI, AKI, stroke, and tracheotomy).

### 2.3 Re-Exploration Procedures

The technique used for mediastinal re-exploration in the CICU was similar to what has been conventionally used in the OR. At our center, each CICU subunit contains 4 to 5 beds separated by curtains. A sterile environment was maintained in our CICU with the aid of a team of scrub nurses. The surgical team was composed of one dedicated surgeon and a CICU nurse with training in OR techniques and occasionally a surgeon’s assistant. All team members followed identical sterilization techniques in both the CICU and the OR. The operating site was prepared with povidone-iodine solution and sterile drapes were used to separate the operating field. The procedure was performed under general anesthesia with an attending anesthetist present throughout the procedure. Heart rate, rhythm, blood pressure, and core temperature were continuously monitored in each patient. For cases with continuous diffuse bleeding that could not be managed surgically, the patient’s sternum was left open with only the skin closed. The sterile packing used was removed once the patient was stabilized.

All patients received routine prophylactic antibiotics with intravenous cephalosporins before the surgical procedure. An additional dose was administered if the operation lasted longer than 4 hours. With reopening in OR, prophylactic antibiotics of 1.5 g cefuroxime were administered to each patient. However, third-generation cephalosporins were applied for patients when reoperation was performed in CICU. Surgical wounds were dressed in a sterile fashion and remained in place for 48 hours to minimize SWI.

### 2.4 Follow-Up

Routine evaluation of the patients’ general health status was conducted once a year by telephone contact after December 2019. If patients passed away at the time of telephone contact, the date and cause of death was obtained from relatives.

### 2.5 Statistical Analysis

SPSS 25 software (IBM Corp, Chicago, IL, USA) was used for statistical analysis. Continuous variables were expressed as mean ± standard deviation or median (interquartile range) based on whether the variables were normally distributed (with non-normal distribution in the Shapiro-Wilk test variables). Students’ *t* test was used to compare normally distributed continuous variables between groups, and the Wilcoxon rank-sum test was used for non-normally distributed continuous variables. Categorical data were presented as frequency and percentage. The Chi-squared or Fisher’s exact test was used to compare categorical variables between groups, when appropriate. To examine whether re-exploration in the CICU was an independent risk factor for postoperative major complications, a univariate logistic regression analysis was used to identify possible risk factors which were than examined by multivariate analysis. We used Kaplan-Meier methods and Cox proportional hazard regression to assess the impact of reoperation in the CICU for both the 30-Day mortality and long-term mortality. All variables with a *p *value less than 0.2 in the univariate analysis were included in the multivariate analysis model or the Cox proportional hazards model. A *p* value less than 0.05 was considered statistically significant.

## 3. Results

Seventy-two patients (1.4% of all screened patients) including 21 who received a re-operation in the CICU and 51 in the OR were eventually selected for further analysis. The mean age of selected patients was 60.0 ± 15.1 years. Fifty-two (72.2%) were male. Forty-six (63.9%) patients received an elective operation, 22 (30.6%) received an emergency operation (within 24 h of hospital admission with cardiac symptoms) and 4 (5.6%) received an urgent operation (upon hospital admission due to uncontrolled cardiac symptoms).

As presented in Table 1, there was no significant difference in baseline characteristics between two groups. The average patient age was slightly higher in the CICU group. More female patients underwent re-explorations in the OR. The incidence of hypertension, diabetes, and stroke, the preoperative left ventricular ejection fraction, and the use of anticoagulant therapy prior to surgery were comparable between the two groups. The average international normalized ratio was relatively higher in the OR group (1.1 ± 0.3 vs 1.0 ± 0.1; *p* = 0.016).

**Table 1.** **Patient characteristics**.

Variables		Total	CICU	OR	*p*
		(n = 72)	(n = 21)	(n = 51)	
Demographic data					
	Age (year)	60.0 ± 15.1	62.8 ± 12.6	58.8 ± 15.9	0.319
	Male (%)	52 (72.2)	17 (81.0)	35 (68.6)	0.571
	BMI (kg/$m^{2}$)	23.0 ± 3.6	23.5 ± 3.1	22.7 ± 3.8	0.436
Medical history					
	Hypertension (%)	40 (55.6)	13 (61.9)	27 (52.9)	0.487
	Diabetes mellitus (%)	11 (15.3)	2 (9.5)	9 (17.6)	0.491
	Chronic dialysis use (%)	2 (2.8)	2 (9.5)	0 (0)	0.082
	Cerebrovascular disease (%)	9 (12.5)	3 (14.3)	6 (11.8)	>0.999
	Marfan syndrome (%)	1 (1.4)	0 (0)	1 (2.0)	>0.999
	Redo cardiac surgery (%)	13 (18.1)	4 (19.0)	9 (17.6)	>0.999
LVEF (%)		50.6 ± 9.5	51.8 ± 11.3	50.1 ± 8.6	0.518
Preoperative anticoagulant therapy (%)		22 (30.6)	5 (23.8)	17 (33.3)	0.425
Preoperative laboratory data					
	WBC (${\mathrm{10}}^{9}$/L)	7.6 ± 3.9	8.6 ± 3.9	7.2 ± 3.9	0.179
	NEU (%)	62.9 (53.1, 81.8)	79.0 (58.1, 88.4)	60.8 (49.7, 76.7)	0.152
	Hemoglobin (g/L)	124.8 ± 23.4	124.4 ± 24.0	125.0 ± 23.3	0.920
	SCr (μmol/L)	73.0 (64.1, 86.3)	86.5 (67.5, 121.8)	71.5 (62.5, 83.0)	0.113
	Platelet (${\mathrm{10}}^{9}$/L)	155.4 ± 57.7	146.2 ± 63.0	159.0 ± 55.8	0.414
	INR	1.1 ± 0.2	1.0 ± 0.1	1.1 ± 0.3	0.016
	PT (s)	11.9 (11.2, 13.0)	12.1 (11.1, 12.9)	11.9 (11.2, 13.1)	0.367
	APTT (s)	28.5 (26.6, 31.3)	27.4 (26.2, 30.1)	28.8 (26.6, 32.7)	0.392
	CRP (mg/L)	3.5 (2.3, 11.5)	3.8 (1.9, 23.1)	3.5 (2.3, 7.0)	0.308
	PCT (ng/mL)	0.04 (0.02, 0.10)	0.04 (0.03, 0.66)	0.04 (0.02, 0.13)	0.394

CICU, cardiac intensive care unit; OR, operating room; BMI, body mass index; LVEF, left ventricular ejection fraction; WBC, white blood cells; NEU (%), percentage of neutrophils; SCr, serum creatinine; INR, international standardized ratio; PT, prothrombin time; APTT, activated partial prothrombin time; CRP, c-reactive protein; PCT, procalcitonin.

Table 2 summarizes the data from the initial cardiac surgery. Two (2.8%) patients received coronary artery bypass, 32 (44.4%) received valve operations, 8 (11.1%) received combined valve and bypass grafting, and 24 (33.3%) patients received aortic operations. The remaining 6 patients (8.3%) received congenital operations, pericardiectomy, or resection of a left ventricular aneurysm, respectively. Additional operative variables including mean cardiopulmonary bypass (CPB) time and cross-clamp time were comparable between two groups. The mean CPB time was significantly prolonged in the CICU group (*p* = 0.037).

**Table 2.** **Initial cardiac surgical features**.

Variables		Total	CICU	OR	*p*
		(n = 72)	(n = 21)	(n = 51)	
Surgical status					0.748
	Elective (%)	46 (63.9)	12 (57.1)	34 (66.7)	
	Urgent (%)	4 (5.6)	1 (4.8)	3 (5.9)	
	Emergency (%)	22 (30.6)	8 (38.1)	14 (27.5)	
Surgical procedure					0.484
	CABG (%)	2 (2.8)	0 (0)	2 (3.9)	
	Valve replace/repair (%)	33 (45.8)	9 (42.9)	24 (47.1)	
	CABG + Valve replace/repair (%)	8 (11.1)	1 (4.8)	7 (13.7)	
	ATAAD surgical repair (%)	25 (34.7)	10 (47.6)	15 (29.4)	
	Other* (%)	4 (5.6)	1 (4.8)	3 (5.9)	
Operation time (min)		335.0 (261.3, 447.5)	370.0 (270.0, 487.5)	315.0 (250.0, 435.0)	0.268
CPB time (min)		181.8 ± 71.1	211.4 ± 70.4	170.6 ± 68.8	0.037
Aortic cross clamp time (min)		133.0 ± 58.1	155.7 ± 58.4	124.5 ± 56.2	0.052
Intraoperative blood loss (mL)		1300.0 (800.0, 2000.0)	1600.0 (1050.0, 2300.0)	1200.0 (800.0, 1525.0)	0.984
Intraoperative transfusion PRBCs (mL)		1112.5 (500.0, 2287.5)	1500.0 (525.0, 2827.5)	1000.0 (400.0, 2000.0)	0.870

CICU, cardiac intensive care unit; OR, operating room; CABG, coronary artery bypass grafting; ATAAD, acute type A aortic dissection; CPB, cardiopulmonary bypass; PRBC, packed red blood cells. *Other includes congenital operation, pericardiectomy, or resection of a left ventricular aneurysm.

Next, we examined and compared the intra-reoperation variables between two groups. Overall, the peak VIS was 47.0 ± 27.4 (51.1 ± 32.2 in the CICU group vs 44.9 ± 25.0 in the OR group, *p* = 0.472). 65 re-explorations (90.3%) were performed within 12 h of the primary operation. The median blood loss volume from initial chest closure to re-exploration was 1100 mL. Before re-exploration, the mean systolic blood pressure was 89.4 ± 9.6 mmHg (88.8 ± 13.4 in the CICU group vs 89.7 ± 7.3 in the OR group, *p* = 0.818), the mean arterial pressure was 67.1 ± 9.6 mmHg (63.9 ± 10.3 in the CICU group vs 68.7 ± 9.0 in the OR group, *p* = 0.122), and the mean central venous pressure was 12.1 ± 4.4 ${\mathrm{cmH}}_{2}$O (13.7 ± 5.4 in the CICU group vs 11.4 ± 3.6 in the OR group, *p* = 0.152). The average serum lactate concentration was 5.5 ± 4.1 mmol/L (6.7 ± 4.9 in the CICU group vs 5.0 ± 3.6 in the OR group, *p *= 0.234); 68 patients (94.4%) had lactate levels higher than 2 mmol/L. The mean hemoglobin level was 74.4 ± 15.4 g/L, and 51 patients (79.8%) had hemoglobin levels lower than 70 g/L (Table 3). No significant differences were found between groups. No patients required CPB support or delayed sternal closure. None of the patients in the CICU group were subsequently transferred to the OR for further re-exploration.

**Table 3.** **Variables between completion of initial cardiac surgery and reoperation**.

Variables	Total	CICU	OR	*p*
	(n = 72)	(n = 21)	(n = 51)	
Hours from completion of initial cardiac surgery to reoperation	3.9 ± 3.0	3.8 ± 3.9	4.0 ± 2.8	0.874
VIS	47.0 ± 27.4	51.1 ± 32.2	44.9 ± 25.0	0.336
Systolic blood pressure (mmHg)	89.4 ± 9.6	88.8 ± 13.4	89.7 ± 7.3	0.818
Mean arterial pressure (mmHg)	67.1 ± 9.6	63.9 ± 10.3	68.7 ± 9.0	0.122
Hemoglobin (g/L)	74.4 ± 15.4	70.0 ± 11.4	76.2 ± 16.5	0.246
Central venous pressure (${\mathrm{cmH}}_{2}$O)	12.1 ± 4.4	13.7 ± 5.4	11.4 ± 3.6	0.152
Serum lactate (mmol/L)	5.5 ± 4.1	6.7 ± 4.9	5.0 ± 3.6	0.234
Drainage volume (mL)	1100.0 (650.0, 1550.0)	1050.0 (675.0, 1935.0)	1200.0 (450.0, 1550.0)	0.604
Reopening operation time (min)	121.6 ± 55.0	117.9 ± 37.2	123.0 ± 60.3	0.740
Blood loss of reoperation (mL)	800.0 (500.0, 1225.0)	1050.0 (775.0, 1575.0)	700.0 (475.0, 1050.0)	0.426

CICU, cardiac intensive care unit; OR, operating room; VIS, Vasoactive-Inotropic Score; PRBC, packed red blood cells.

**Fig. 1.**
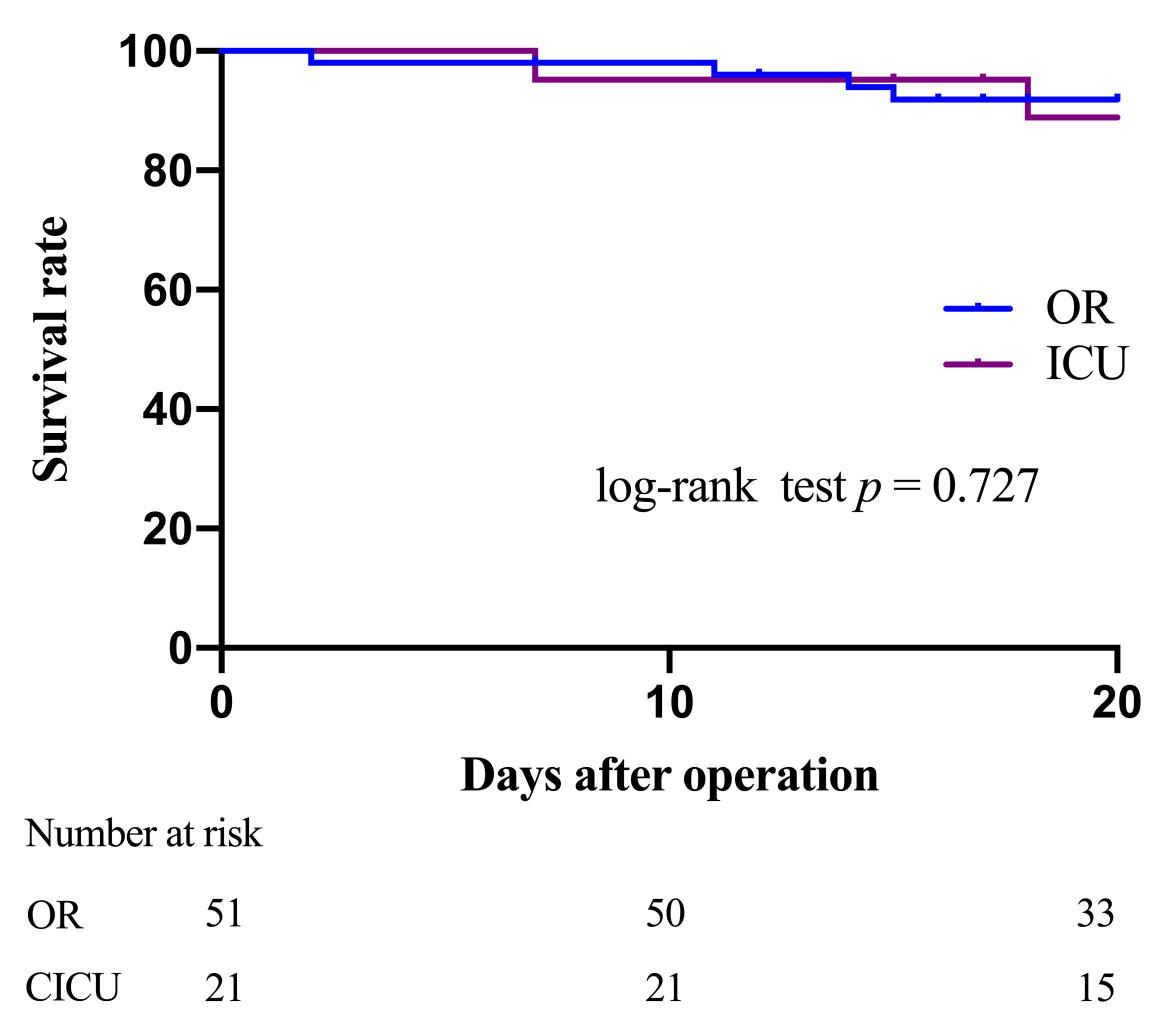
**Kaplan-Meier curves for 30-Day mortality in the two group**. OR, operation room; CICU, cardiac intensive care unit.

As shown in Table 4, there was no significant difference in post exploration laboratory test results between two groups. In addition, the average CICU stay and hospital stay was comparable between two groups. The incidence of post re-exploration adverse events including SWI, pneumonia, prolonged ventilation, AKI, new-onset hemodialysis, new-onset atrial fibrillation and tracheotomy were similar between two groups. Hospital costs were significantly lower in the CICU group. However, the cost for reoperation was not significantly different between the two groups. In addition, there was no significant difference in the 30-Day mortality between the CICU group and the OR group (14.3% vs 11.8%). Kaplan-Meier curves revealed no significant difference in 30-Day mortality between the two groups (log-rank *p* = 0.727, Fig. 1). After adjusting for confounders, the hazard ratios for reoperation conducted in the CICU (hazard ratios: 1.304, 95% confidence interval (CI): 0.325–5.232, *p *= 0.708) were not significantly associated with poor short-term survival.

**Table 4.** **Postoperative laboratory data and outcomes**.

Variables		Total	CICU	OR	*p*
		(n = 72)	(n = 21)	(n = 51)	
Postoperative day 1 laboratory data					
	WBC (${\mathrm{10}}^{9}$/L)	11.7 ± 8.2	14.3 ± 13.5	10.6 ± 4.5	0.232
	NEU (%)	86.3 ± 5.6	87.3 ± 5.6	85.9 ± 5.7	0.359
	Hemoglobin (g/L)	85.5 ± 18.4	83.1 ± 12.6	86.5 ± 20.3	0.484
	SCr (μmol/L)	94.0 (72.0, 147.5)	94.0 (76.5, 217.5)	91.0 (72.0, 121.0)	0.236
	Platelet (${\mathrm{10}}^{9}$/L)	83.3 ± 38.7	76.0 ± 25.1	86.1 ± 42.8	0.323
	INR	1.5 ± 0.4	1.5 ± 0.4	1.5 ± 0.4	0.984
	PT (s)	16.2 ± 4.0	16.5 ± 4.1	16.1 ± 4.1	0.720
	APTT (s)	37.2 (32.1, 59.6)	40.2 (33.9, 67.3)	36.8 (31.8, 59.0)	0.939
	CRP (mg/L)	122.0 ± 53.4	124.5 ± 54.9	120.9 ± 53.3	0.801
	PCT (ng/mL)	2.0 (0.5, 7.9)	1.3 (0.4, 3.9)	2.3 (0.5, 10.5)	0.463
Postoperative characteristics					
	AKI (%)	33 (45.8)	10 (47.6)	23 (45.1)	0.845
	ECMO (%)	2 (2.8)	1 (4.8)	1 (2.0)	>0.999
	IABP (%)	3 (4.2)	0 (0)	3 (5.9)	0.551
	Pneumonia (%)	24 (33.3)	8 (38.1)	16 (31.4)	0.582
	Sputum culture (+) (%)	26 (36.1)	9 (42.9)	17 (33.3)	0.444
	Blood culture (+) (%)	8 (11.1)	2 (9.5)	6 (11.8)	>0.999
	Catheter head culture (+) (%)	4 (5.6)	1 (4.8)	3 (5.9)	>0.999
	Stroke (%)	9 (12.5)	3 (14.3)	6 (11.8)	>0.999
	Paraplegia (%)	1 (1.4)	1 (4.8)	0 (0)	0.292
	Prolonged ventilation >48 hours (%)	38 (52.8)	13 (61.9)	25 (49.0)	0.320
	Reintubation (%)	17 (23.6)	4 (19.0)	13 (25.5)	0.762
	New-onset atrial fibrillation (%)	22 (30.6)	4 (19.0)	18 (35.3)	0.174
Major complications (%)		18 (25.0)	5 (23.8)	13 (25.3)	0.881
	Sternal wound infections (%)	4 (5.6)	1 (4.8)	3 (5.9)	>0.999
	New-onset hemodialysis (%)	12 (16.7)	4 (19.0)	8 (15.7)	>0.999
	Tracheotomy (%)	6 (8.3)	0 (0)	6 (11.8)	0.171
30-Day mortality (%)		9 (12.5)	3 (14.3)	6 (11.8)	>0.999
CICU days		6.0 (3.0, 13.0)	5.0 (4.0, 9.5)	7.0 (3.0, 13.0)	0.775
Length of stay (day)		26.0 ± 13.7	24.2 ± 9.7	26.8 ± 15.1	0.466
Reoperation costs (¥)		25437.2 ± 7453.6	24398.5 ± 6102.6	26693.7 ± 8469.4	0.582
Hospital costs (¥)		235450.9 ± 145362.0	210911.3 ± 62689.6	245489.7 ± 167530.2	0.018

CICU, cardiac intensive care unit; OR, operating room; WBC, white blood cells; NEU (%), percentage of neutrophils; SCr, serum creatinine; INR, international standardized ratio; PT, prothrombin time; APTT, activated partial prothrombin time; CRP, c-reactive protein; PCT, procalcitonin; AKI, acute kidney injury; ECMO, extracorporeal membrane oxygenation; IABP, intra-aortic balloon pump.

In the univariate analysis, surgical status consisting of emergency and non-emergency, surgical procedure consisting of acute type A aortic dissection (ATAAD) surgery and non-ATAAD surgery were included in the analysis. Eventually, seven parameters were included in the multivariate logistic analysis model. The analysis suggested that emergent surgery, ATAAD surgery, initial cardiac surgery CPB time, and reopening operation time were independent risk factors for developing postoperative major complications. Nevertheless, reoperation conducted in the CICU (odds ratio: 0.958, 95% CI: 0.342–3.071, *p *= 0.806) was not identified as a risk factor for postoperative major complications (Table 5).

**Table 5.** **Multivariate analysis of risk factors for postoperative major complications**.

Variables	Odds ratio	95% CI	*p*
Age	1.242	0.995–1.201	0.064
Redo cardiac surgery	1.285	0.238–8.434	0.805
Preoperative anticoagulant therapy	1.846	0.643–6.174	0.482
Emergent surgery	8.420	2.569–28.548	0.002
ATAAD surgery	4.162	1.009–9.996	0.032
Initial cardiac surgery CPB time	1.021	1.005–1.126	0.003
VIS	1.044	0.972–1.135	0.146
Reoperation in CICU	0.958	0.342–3.071	0.806
Reopening operation time	1.141	1.007–1.287	0.024

CI, confidence interval; ATAAD, acute type A aortic dissection; CPB, cardiopulmonary bypass; CICU, cardiac intensive care unit.

**Fig. 2.**
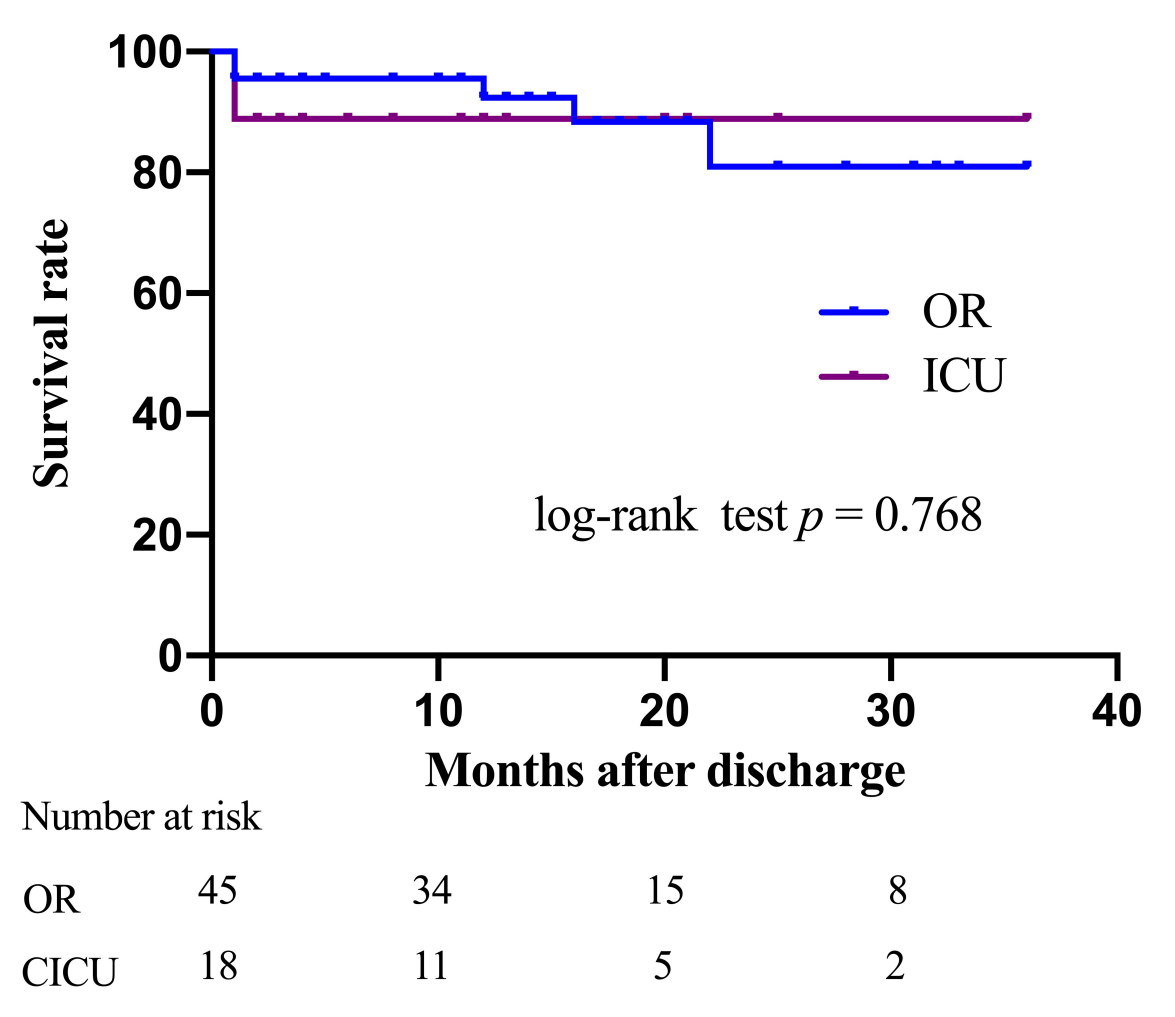
**Kaplan-Meier curves for long-term mortality in the two group**. OR, operation room; CICU, cardiac intensive care unit.

By January 2022, all patients had been followed for a median of 12 months. No SWI event was identified after discharge. Four patients from the OR group died during the follow-up period. However, Kaplan-Meier curves revealed no significant difference in cumulative survival rate between the groups (log-rank *p* = 0.768, Fig. 2). Multivariate Cox analysis for mortality revealed that reoperation conducted in the CICU (hazard ratios: 1.278, 95% CI: 0.224–6.697, *p *= 0.772) was not a significant risk factor after adjusting for other major clinical factors.

## 4. Discussion

In this study, we demonstrated that conducting reoperations in the CICU did not result in additional risks such as SWI, hospital stay and mortality as compared to surgeries conducted in the OR. These results support an alternative approach when post-cardiac reoperation is required. To the best of our knowledge, this was the first contemporaneous study describing the outcomes of re-explorations for bleeding performed at different locations in the hospital.

Limited studies had been published to resolve the debate as to whether routine mediastinal re-explorations after cardiac operations can be safely conducted in the CICU. Most previous studies failed to compare the results to the conventional OR setting. As a result, the most efficacious strategy for mediastinal re-exploration remained unidentified. Conducting re-exploration in the CICU also has some advantages such as avoiding patient transfer under unstable hemodynamic conditions. More cardiac intensive care units now have sterile environments and the availability of additional life-saving equipment which are equivalent to that found in the OR. Our results showed that reoperations conducted in the CICU can be performed safely and effectively.

Recent studies suggested that the incidence of re-exploration for bleeding ranges from 2.2% to 5.9% [4, 5, 10, 11]. A total of 1.4% of patients were re-explored in this cohort, which is below the lower end of the range reported in the literature. However, previous results may have been confounded by the inclusion of reoperations performed for emergent conditions. In this study, we excluded patients who received mediastinal re-exploration due to cardiac arrest and cardiac tamponade, which resulted in a lower incidence of reoperations.

It is important to point out that the morbidity and mortality reported in this study for re-explorations was higher than those who received primary cardiac surgery. Based on our experience, this difference was likely attributed to the hemodynamic consequences of excessive bleeding rather than the re-exploration surgery itself. In this study, 5.6% of all patients experienced SWI and 12.5% of patients died after cardiac reoperations for bleeding; which is consistent with other studies [4, 6, 7, 10, 12, 13]. The lower incidence of SWI and mortality reported in some studies might be attributed to the exclusion of aortic dissection patients in their analyses. Reoperation procedures could aggravate the inflammatory response and lead to respiratory or renal dysfunction. The elevated reintubation and new onset dialysis rate after reoperation surgery in our study might be attributed to the augmented inflammatory response.

A primary concern for conducting re-explorations in the CICU is the fear of SWI; which remains a life-threatening complication after cardiac operations. Postoperative hemorrhage, prolonged operation and CPB times as well as hospital stay before the re-operation, internal mammary artery harvesting, immunocompromised states, and diabetes mellitus are considered as predisposing factors for SWI. Early postoperative re-exploration has also been identified as a predisposing factor for SWI [14]. Our data demonstrated that the occurrence of SWI was comparable between the CICU and the OR. This may due to the fact that we employed similar aseptic techniques in the CICU as in OR. Furthermore, only attending cardiac surgeons or senior trainees were eligible to conduct the re-exploration, accompanied by OR trained nursing staff. The mortality rates and occurrence of other postoperative complications were also comparable between two groups. Moreover, as suggested by the logistic regression analysis, the location where the re-exploration was performed was not an independent risk factor for major postoperative complications. This study showed that planned re-explorations conducted in the CICU are associated with comparable outcomes, similar to those that are performed in the OR for bleeding following cardiac surgery.

Hemorrhagic shock is one of the major causes of death in trauma patients [15], and is also commonly seen after cardiac surgery [16, 17, 18]. The main pathophysiological change in hemorrhagic shock is sudden reduction of effective circulating volume which leads to tissue hypoperfusion, increased anaerobic metabolism, lactic acidosis, reperfusion injury, endotoxin translocation, and ultimately leads to multiple organ dysfunction [19]. Rapid recognition, fluid resuscitation, and use of vasopressor drugs are essential in treating hypovolemic shock. A previous study indicated that patients received re-exploration for bleeding after cardiac surgery were at higher risk of experiencing adverse outcomes and this risk was further increased if the time to re-exploration was 12 h or longer [20]. Therefore, prompt re-exploration for bleeding which occurs after cardiac surgery is strongly recommended.

This study has some limitations. First, this was a retrospective study conducted in a single center with a small cohort. Second, the indication for re-exploration was not defined in advance. Third, the similar incidence of adverse events in two groups might be due to the limited number of patients which reduces the statistical power for risk factor analysis. Finally, relatively few patients received re-explorations in the CICU in this study sample (29.2%) which limited statistical modeling efforts and empiric data analysis. Therefore, further prospective multicenter studies are needed to better identify the most effective strategies to improve the prognosis of patients who undergo reoperation for bleeding following cardiac surgery.

## 5. Conclusions

In conclusion, our study found that planned re-exploration for bleeding after cardiac surgery can be safely and effectively conducted in the CICU. The CICU can serve as an alternative site to the OR to re-explore these high-risk patients.

## Data Availability

The datasets used and/or analyzed during the current study are available from the corresponding author on reasonable request.

## Abbreviations

OR, operation room; CICU, cardiac intensive care unit; SWI, sternal wound infection; VIS, Vasoactive-Inotropic Score.
